# Infant neural processing of mother’s face is associated with falling reactivity in the first year of life

**DOI:** 10.1016/j.dcn.2024.101502

**Published:** 2024-12-30

**Authors:** Silvia Rigato, Manuela Stets, Henrik Dvergsdal, Karla Holmboe

**Affiliations:** a Centre for Brain Science, Department of Psychology, University of Essex, UK; b Business Administration Programme, Nord University, UK; c School of Psychological Science, University of Bristol, UK

**Keywords:** ERPs, Face processing, Infancy, Infant temperament

## Abstract

It is well established that faces evoke a distinct neural response in the adult and infant brain. Past research has focused on how the infant face-sensitive ERP components (N290, P400, Nc) reflect different aspects of face processing, however there is still a lack of understanding of how these components reflect face familiarity and how they change over time. Further, there are only a few studies on whether these neural responses correlate with other aspects of development, such as infant temperament. In this longitudinal study (N∼60), we recorded infant visual ERPs in response to mother and stranger face stimuli at 4, 6 and 9 months of age. Our results showed that, compared to a stranger face, the mother face evoked a larger N290 at 4 months and a larger P400 at 6 months. At 9 months, no difference was found between mother and stranger faces. However, at 9 months we found that the P400 and Nc amplitudes evoked by the mother face were associated with infant falling reactivity. We conclude that the neural responses associated with the processing of faces, and specifically the face of the mother, are related to the development of infant individual characteristics.

## 1 Introduction

It is well documented that newborns tend to orient preferentially towards faces (Johnson et al., 1991), and prefer looking at faces that are socially more relevant to them. These include faces with direct gaze (Farroni et al., 2002), faces displaying happy emotional expressions (Farroni et al., 2007), and the infant’s own mother’s face (Field et al., 1984, Bushnell et al., 1989); Pascalis, deSchonen, Morton, Deruelle, & Fabre-Grenet, 1995). Event-related potential (ERP) studies have been key to shedding light on face processing abilities in the first months of life (e.g., Halit, de Haan, & Johnson, 2003). Infant ERP studies have suggested that the processes underlying the well-known adult face-sensitive N170 component (e.g., Bentin et al., 1996) are evident as two distinct occipito-temporal components that are elicited within the first 500 ms after the presentation of a face stimulus; a negative peak around 290 ms (N290) and a positive deflection around 400 ms (P400) (Halit et al., 2003). These components are thought to represent the N170 infant precursors because of their strong face sensitivity across the first year of life (e.g., (de Haan et al., 2002; de Haan et al., 2002; Halit et al., 2003; Halit et al., 2003).

In a study with 6-month-old infants, Balas et al. (2010) modulated face orientation (upright vs. inverted) in a within-subjects design, varying face familiarity (mother vs. stranger) across participants. The authors were interested in whether the P400 component exhibited differential processing for upright and inverted faces as a function of face familiarity. They found a larger P400 amplitude for mother-inverted vs. mother-upright faces but no difference for stranger-inverted vs. stranger-upright faces. Balas et al. (2010) therefore concluded that the neural mechanisms underlying face processing at 6 months of age reflect selectivity learned from experience with particular faces.

In addition to the N290 and P400, ERP studies have identified another component that shows sensitivity to faces; the Negative central (Nc) component, which peaks over frontocentral electrodes between 400 and 800 ms after stimulus onset (Parise et al., 2010, Marinović et al., 2014, Guy et al., 2016). The Nc has been defined as an obligatory attention response, i.e., associated with the obligatory recruitment of attention to a visual stimulus (Courchesne et al., 1981, Karrer and Ackles, 1987, Nelson, 1996, Nelson and Collins, 1991) and, of particular relevance for the present study, it has also been found to be enhanced in response to the mother’s face (vs. an unfamiliar face) in 6-month-old infants (de Haan and Nelson, 1997). Altogether, this suggests that the Nc reflects processes related to attention and stimulus recognition for information stored in long-term memory. Snyder et al. (2002) also proposed that a familiar stimulus such as the mother’s face evokes either more, or a different kind of, attention than an unfamiliar stimulus.

In a longitudinal study with infants between 4 and 12 months of age, Webb and colleagues (Webb et al., 2005) recorded the Nc and two additional ERP components (Pb, PSW) over frontocentral sites in response to images of familiar and unfamiliar stimuli (i.e., mother and stranger’s faces, and favourite and novel toys). They found differences in ERP developmental trajectories for stimulus type and familiarity, observing that the Nc did not respond in a uniform way to familiar stimuli across ages. The authors therefore suggested that changes in ERP responses to mother and stranger faces in the first year of life may represent the effects of developmental changes in cognitive abilities, repeated testing, or differences in novelty preference. Evidence from studies with older children further suggests that changes in ERP responses to mother and stranger faces might also be related to differences in temperament traits and attachment (Carver et al., 2003).

According to Shiner et al. (2012), temperament traits are early emerging basic dispositions in the domains of activity, affectivity, attention, and self-regulation, resulting from complex interactions of genetic, biological, and environmental factors across time. Self-regulation indicates those “processes that serve to modulate reactivity” (Rothbart et al., 2011), p.442) and includes sensorimotor activities and voluntary behaviors that promote appropriate responses to situational demands (Van den Bergh and Mennes, 2006); for a systematic review, see (Pinto and Figueiredo, 2023). Among these, for example, orienting serves as a major self-regulatory mechanism as infants who can quickly disengage from distressing objects are less susceptible to negative affect and easier to soothe (Crockenberg and Leerkes, 2004, Morales et al., 2005).

There exist a few evidences that indicate how self-regulatory behaviors are associated to face processing in infants. An ERP study found that proximity-seeking behaviour during interactions with the mother was correlated with the Nc component in response to the mother’s face in 6-month-old infants (Swingler et al., 2007). Specifically, this study reported a smaller Nc amplitude to the mother’s face vs a stranger’s face in those infants who showed a greater number of attachment-like behaviours that secured close proximity and continued interaction with the mother. A subsequent study from the same research group (Swingler et al., 2010) reported that 6-month-old infants’ distress and visual search for mother during separation correlated with face-related ERPs. These associations were different for mother and stranger faces so that a higher level of distress was specifically associated with processing of the mother’s face (larger P400 amplitude; larger Nc amplitude on the left sites and smaller Nc amplitude on the right sites; longer Nc latency), while visual search for mother was associated with processing of the stranger’s face (longer P400 and Nc latencies). Overall these findings provide support for a relationship between the developing neural face processing system and behaviours associated with social interactions within the infant-mother dyad.

Another piece of evidence pointing to the existence of associations between how infants process their mother’s face and socio-emotional development comes from our own behavioural study (Rigato et al., 2023). In this study, we investigated the longitudinal trajectory of infant visual preference for the mother’s face from 2 weeks to 9 months of age and how changes in such preference relate to individual temperament characteristics of emotional reactivity (negative affect, distress, falling reactivity). While we only found weak associations between looking time to the mother’s face and distress (and none with negative affect), we found that looking longer at the mother’s face at 6 months was significantly associated with falling reactivity at 9 months of age. We interpreted this finding as an indication that those infants who spend more time looking at their mother (vs. a stranger’s face) develop better regulatory skills later on. This could be because when infants experience distress, they tend to orient towards their mothers – who would then soothe them – as a regulatory mechanism that allows them to reduce distress (Kopp, 1989).

In the present work, we report findings from another study conducted with the same cohort of infants as in Rigato et al. (2023). Here, we recorded ERPs over clusters of occipitotemporal and frontal electrodes in infants at 4, 6 and 9 months while they watched images of their own mother’s face and a stranger’s face. We were interested in i) observing developmental changes of the face sensitive components N290, P400 and Nc, ii) identifying time points where such components showed significant differences between the face stimuli; and iii) how these neural responses correlated with infant temperament. While little is known about how the neural correlates elicited by (familiar and novel) faces change over time, based on the existent evidence we expected to observe the face-sensitive components N290, P400 and Nc at each time point (though in Balas et al. (2010) study the N290 was absent). We also expected a larger Nc for the mother’s face vs. stranger’s face, at least at 6 months, as reported in de Haan and Nelson (Nelson, 1996), and a significant difference between the face stimuli for the P400 (larger for the mother’s face), as this component has been suggested to reflect experience with particular faces (see Balas et al. (2010), though the comparison in their study involved upright (i.e. familiar) and inverted (i.e. novel) faces).

In terms of longitudinal associations with infant temperament, based on our behavioural findings in Rigato et al. (2023), we focused on the dimension of Falling Reactivity. This construct describes to what extent an individual is capable of recovering from a high level of distress or positive excitement, as such, in the current context it refers to the infant’s ability to self-regulate (Gartstein and Rothbart, 2003). Given the relationship found between infants’ looking time to the mother’s face at 6 months and falling reactivity at 9 months in our behavioural study (Rigato et al., 2023), we hypothesised a longitudinal association between neural processing of the mother’s face at 6 months and falling reactivity at 9 months. However, given that EEG has the potential to uncover processes before they can be observed in overt behaviour, we reasoned that earlier associations – as well as within age associations – may also be observed. Finally, based on Swingler et al., 2007, Swingler et al., 2010, we expected these associations to involve the P400 and Nc components.

## 2 Methods

### 2.1 Participants

A total of 81 families were recruited from the University of Essex Babylab database and through events for expectant parents at Colchester General Hospital and other local venues in Essex, UK. Ethical approval was gained from the National Research Ethics Committee, London-Hampstead branch, United Kingdom (15/LO/0478; 17/03/2015) and from the University of Essex Ethics Sub-Committee. All methods were performed in accordance with the Declaration of Helsinki. Informed written consent was obtained from the parents of the infants prior to data collection at each testing session.

The current manuscript reports on data from a longitudinal study investigating attention and social skills in the first year of life using multiple methods (behavioral, neuroimaging and questionnaires), and consisting of one pre- and four postnatal assessment points. The postnatal lab sessions were scheduled at the following ages: 2 weeks, 4 months, 6 months, and 9 months after birth. While previously published articles include further details on the participant sample and other longitudinal data from these infants (Rigato et al., 2020, Rigato et al., 2022, Rigato et al., 2023, Rigato et al., 2024), none includes data from the EEG face processing task reported in this manuscript. Of the 81 recruited families, 16 mothers participated only in the prenatal assessment and could therefore not be included in the current analyses. Additionally, 2 infants suffered from complications at birth, and were excluded from the final sample. The majority of participating infants were born full-term (at 37 weeks of gestation or later; *M* = 40 weeks, 3 days), and only two infants were born between 36 and 37 weeks of gestation. Infants in the included sample had normal birth weight (*M*(*SD*) = 3.5(0.4) Kg; Range = 2.44–4.82 Kg), no complications at birth, and no known health issues (pregnancy, birth and health information was missing for 4 infants). At the time of the prenatal assessment, which occurred around 36 weeks of gestation, mothers were on average 31.2 years of age (*SD* = 4.6); fathers were on average 33.6 years of age (*SD* = 6.1). Mothers had spent an average of 16.8 years in education (*SD* = 3.6) and fathers had spent an average of 15.2 years in education (*SD* = 3.7). The infants’ ethnic backgrounds were mainly White British (*N* = 54; 85.7 %). Three infants were of Other White background (4.8 %), four infants had mixed ethnic backgrounds but did not provide further details (6.4 %), one infant was White British mixed with another ethnic background (1.6 %), and one infant had a Hispanic background (1.6 %).

For this study about 60 infants came to the lab at three time points. Specifically, 63 infants took part in the study at 4 months, 61 at 6 months, and 62 at 9 months. The main criterion for an infant to be included in the final sample was the contribution of at least 10 valid trials per condition (e.g., Kobiella et al., 2008; Leppänen et al., 2007). Across the three sessions, reasons for exclusion from the final analysis were fussiness, movement artefacts, inattentiveness to the screen during stimulus presentation, or an excessive number of channels (>10 %) recording artefactual data. Therefore, data from 25 4-month-olds, 21 6-month-olds, and 22 9-month-old infants had to be excluded (attrition rate in line with previous EEG studies, see (Stets et al., 2012; Van der Velde and Junge, 2020). Consequently, the final sample included in the analyses consisted of 38 4-month-olds (16 females, M age (days): 123.3, SD = 6.6), 40 6-month-olds (12 females, M age (days): 185, SD = 8.2) and 40 9-month-olds (14 females, M age (days): 279.8, SD = 14.9).

### 2.2 Questionnaire measures

Mothers were asked to complete a well-established questionnaire measuring infant temperament, the Infant Behavior Questionnaire–Revised Very Short Form (IBQ-R VSF; (Putnam et al., 2014) as well as two additional scales from the Infant Behavior Questionnaire–Revised (IBQ-R; Gartstein and Rothbart, 2003), i.e., Distress and Falling Reactivity, at 2 weeks, 4 months, 6 months, and 9 months. The link to the online questionnaire was sent via email to those mothers who had confirmed attendance for a scheduled lab test session the following day, in an effort to keep the time difference between test session and questionnaire completion as short as practically possible.

Given our research questions in the present study, we report on scores of the Falling Reactivity scale (13 items; e.g., “When put down for a nap, how often did your baby settle down quickly?”) in our main analyses (although additional correlation analyses involving the Negative Affect and Distress scales can be found in Table S3 and Table S4, Supplementary Material). Cronbach’s alpha for the Falling Reactivity scale ranged from.65 to.85 across ages, with a mean alpha of.78. Details of the other scales can be found in Supplementary Methods, Supplementary Material.

### 2.3 Stimuli

During a lab visit prior to childbirth, the expecting mothers had a picture taken of their face using a digital camera. The images showed fully frontal views of a mother’s head and neck with the face showing a friendly expression and a medium-large smile. Mothers were asked to look straight-ahead, directly into the camera. In order to prevent variation in the image material regarding lighting conditions and colorfulness, pictures were taken in front of the same white wall and under the same lighting conditions. Additionally, mothers were asked to remove earrings and to wear a white towel around their neck to ensure a high similarity in the neckline for all images. Subsequently, images were digitally processed using the Adobe Photoshop package, i.e., head-outlines were cut out from their background to eliminate potential shadows and then placed onto a uniform, medium-grey background instead. When presented on the screen, faces had a width of 18.6 cm and a length of 21.2 cm (700 ×800 pixels, respectively), with a visual angle of 17° x 20°.

The stranger face selected for the EEG task for each infant was a mother of another infant participating in the study (this image was kept constant at each visit). Selection criteria for finding an appropriate stranger were that the mothers and, consequently, the infants did not know the other mother and that they looked sufficiently different from each other without being extremely dissimilar. For instance, if a mother was wearing glasses when the image was taken, the respective stranger was selected such that she would be wearing glasses as well. Similarly, if a mother had her mouth closed when smiling, the respective stranger was selected such that she also had her mouth closed.

### 2.4 EEG task procedure

Infants sat on their parent’s lap 60 cm away from a 23-inch computer monitor in a quiet and dimly lit room. Each trial started with a colour cartoon image displayed in the middle of the screen for a duration of either 300, 400 or 500 ms, equally distributed across the trials. Following this, a face stimulus (the mother or the stranger face) replaced the cartoon image for 1000 ms. Sounds were occasionally used to re-direct infants’ attention toward the screen and were played during cartoon presentation. Face stimuli were presented in a random order for a total of 200 trials (100 trials for each stimulus type) or until the infant became fussy and inattentive. The average number of trials considered for the analysis at 4 month was 16.2 (*SD*=6.5) for the mother face and 17.5 (*SD*=6.3) for the stranger face; at 6 months it was 14.2 (SD=4.3) for the mother face and 14.2 (SD=4.5) for the stranger face; and at 9 months it was 13.9 (SD=4.1) for the mother face and 13.1 (*SD*=3.7) for the stranger face.

### 2.5 EEG recording and analysis

**Fig. 1:**
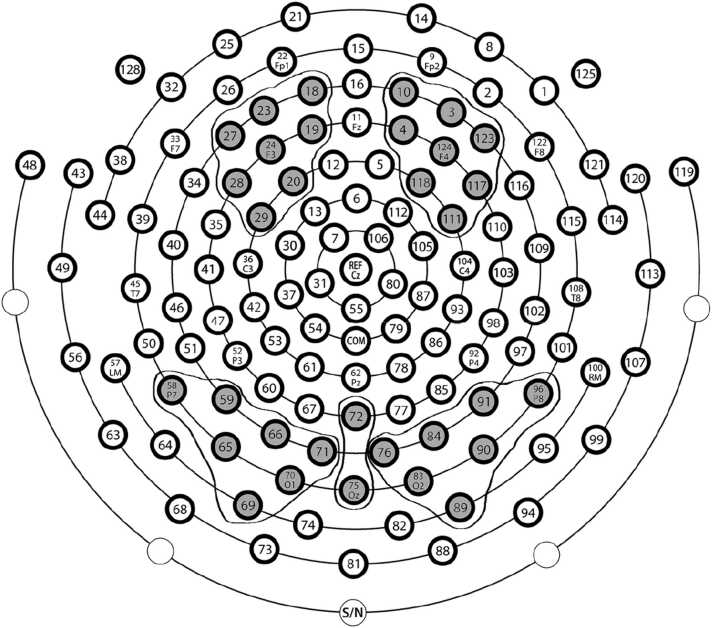
Hydrocel Geodesic Sensor Net configuration. Highlighted in grey are the channels selected for each cluster used in the analyses.

Brain electrical activity was recorded continuously using a Hydrocel Geodesic Sensor Net consisting of 128 electrodes evenly distributed across the scalp (Fig. 1) and referenced to the vertex. EEG was amplified with a 0.1–100 Hz band-pass filter and digitized at 500 Hz. Off-line analysis was conducted using EEGlab / Matlab (version 12.0.2.5b; Delorme and Makeig, 2004). Continuous EEG data were low-pass filtered at 30 Hz using digital elliptical filtering, bad channels were visually inspected and manually marked as bad to be excluded during re-referencing, data were re-referenced to the average potential over the scalp, and segmented in epochs from 100 ms before until 700 ms after stimulus onset. Segments with eye movements and blinks were detected manually and rejected from further analysis. Artefact-free data were then baseline-corrected to the average amplitude of the 100 ms pre-stimulus interval. Finally, individual and grand averages were calculated based on the above-mentioned inclusion criteria.

Statistical analyses of the ERP data targeted the examination of face condition (mother, stranger) over right, left and medial occipitotemporal electrode sites and over right and left frontocentral electrodes. Groups of electrodes were initially selected for analysis based on previous studies of face processing (e.g., de Haan et al., 2002; (Rigato et al., 2010; Gillmeister et al., 2019) and this selection was adjusted following visual inspection to find where the components of interest were maximal (i.e. maximum amplitude of the ERPs) in the present dataset for each age group. For the analyses of the occipitotemporal components (N290 and P400), the electrodes included in the analyses were: 76, 83, 84, 89, 90, 91, 96 (right hemisphere); 72, 75 (medial electrodes); 58, 59, 65, 66, 69, 70, 71 (left hemisphere) (Fig. 1). Waveforms from these electrodes were averaged to create left, right and medial electrode clusters for each condition. For the analyses of the Nc, the following frontocentral electrodes were selected: 3, 4, 10, 111, 117, 118, 123, 124 (right hemisphere); 18, 19, 20, 23, 24, 27, 28, 29 (left hemisphere).

Because of developmental changes in the morphology and latency of the face-sensitive components (de Haan et al., 2003), we defined time windows for each component of interest based on visual inspection of individual peaks at each age. Over the occipitotemporal sites, we looked for the N290 and P400 infant face-sensitive components. At 4 months of age, we observed that the face stimuli elicited both the expected components, however at 6 and 9 months only a P400 was observed. Therefore, the analyses focused on the N290 (240–340 ms time-window) and P400 (400–600 ms time window) for the data collected at 4 months, but only on the P400 component (250–450 ms time window) for the data collected at 6 and 9 months. Over the frontocentral sites, we identified the expected Nc at each age. This was defined as being within the time window 400–600 ms after stimulus onset at 4 and 9 months, and between 250 and 500 ms at 6 months (as an earlier component was identified). The peak amplitudes and latencies of the occipitotemporal ERP components for the mother and stranger face stimuli were compared with a 2×3 ANOVA with face type (mother, stranger) and location (right, left, medial) as within subject factors, while the mean amplitude of the frontocentral Nc was analysed with a 2 × 2 ANOVA with face type (mother, stranger) and electrode cluster (right, left) as within subject factors. Main effects and interaction between factors were included in each analysis. Mean amplitude was used as a conservative measure of the Nc waveform; for the N290 and P400 components peak amplitudes and latencies were used as these are most appropriate when precise peaks can be identified for each individual's set of components.

Following these analyses, a linear mixed model (LMM) was run (SPSS 27) to investigate the developmental trajectories, i.e., an effect of age, for the ERP components that were present at all ages (P400 and Nc). For these analyses, the amplitude of the ERP components was averaged across sites to minimise the number of comparisons and the risk of Type 1 errors. The models included Age, Face type and Age x Face type interaction as fixed factors, and participants as a random factor. Fifty-nine participants were included in the LMM. ERP data for each assessment time point (4, 6 and 9 months) were included for 18 participants, while the other 41 participants had some missing points (from 1 or 2 timepoints). Family income of those participants who contributed to the data at each point of assessment and those for which we had missing points did not differ (p > .124; see Table S1 in the Supplementary Material for information about missing data). Maximum likelihood estimation was used to account for missing data. Results of Little’s missing completely at random test (Little, 1988) suggest that the data in this study were missing completely at random; χ^2^ (18) = 21.607, *p* = .250.

Satterthwaite method for degrees of freedom was used. Finally, longitudinal associations between the amplitude of the ERP components (N290, P400, Nc) evoked by mother and stranger face stimuli at each age and the average score of infant falling reactivity across the first 9 months of life were assessed.

## 3 Results

### 3.1 ERPs evoked at 4 months

**Fig. 2:**
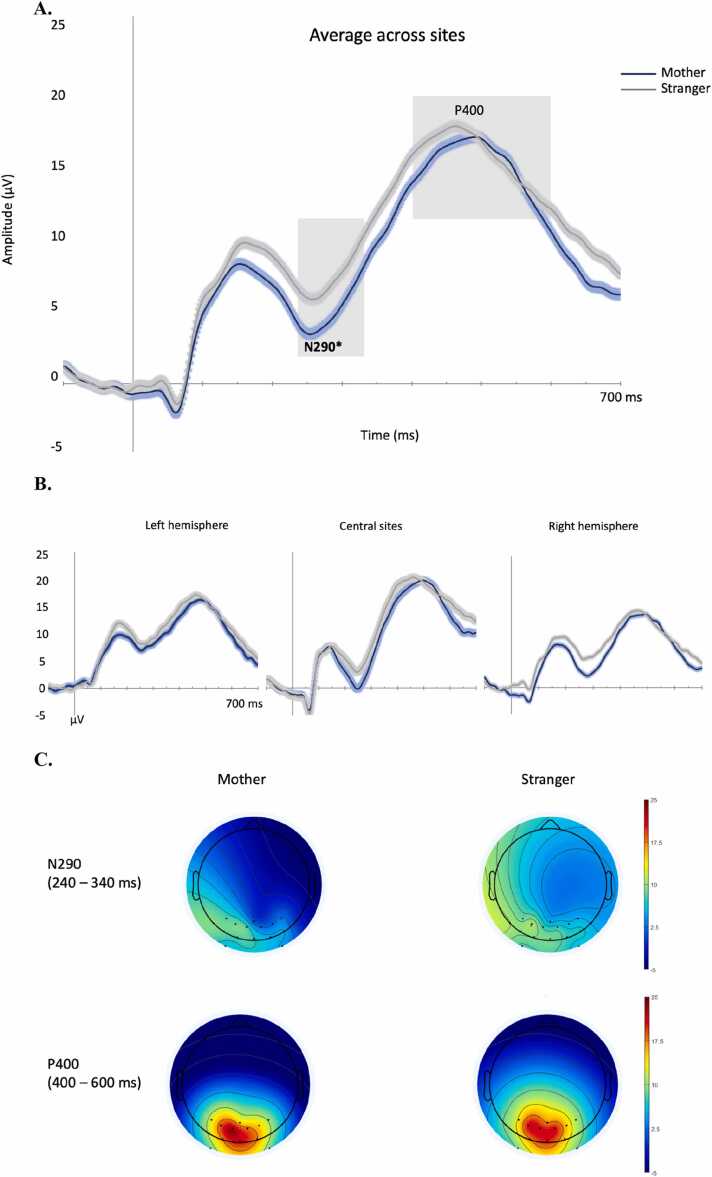
Grand average of the occipitotemporal ERPs evoked by mother and stranger face stimuli in infants at 4 months of age. The time-windows selected for the analyses are highlighted in grey. *main effect of face type p < .05. A.: average of ERPs across sites; B.: ERPs over left, right and central sites; C.: topological maps of the ERPs time window per face type.

At 4 months, mother and stranger face stimuli elicited clear N290 and P400 components over the occipitotemporal sites (see Fig. 2). Statistical analyses revealed that the peak amplitude of the N290 was affected by face type, F(1,37)= 6.448, p = .015, η_p_^2^ = .14, so that it was larger to mother (M = −.29 μV) than to stranger face stimuli (M = 2.43 μV). The N290 amplitude was also affected by location, F(2,74)= 8.048, p < .001, η_p_^2^ = .18 (M right = −0.1 μV; M left = 3.8 μV; M central = −0.5 μV). Paired-sample t tests revealed that the N290 was significantly larger over the right and central sites than the left sites, t(37) = 3.580, p < .001, t(37) = 3.848, p < .001, respectively. The face type x location interaction was not significant for the N290 amplitude (p > .6). The latency of the N290 was affected by location, F(2,74)= 24.722, p < .001, η_p_^2^ = .40, with an earlier peak detected over the central sites (M=262 ms) than the right (M= 287 ms), t(37) = 7.528, p < .001, and the left hemisphere (M=284 ms), t(37) = 5.835, p < .001. The main effect of face type and the interaction with location were not significant for the N290 latency (ps>.32). The analyses of the P400 component showed that its peak amplitude was affected by location only, F(2,74)= 5.809, p = .005, η_p_^2^ = .14, showing larger amplitude over the central sites (M= 24.7 μV) than over the right (M= 21.5 μV), t(37) = 3.675, p < .001, and the left hemisphere (M=19.7 μV), t(37) = 2.020, p = .05. No significant effect of face type or interaction were found (ps>.78). No significant main effects or interaction were found for the P400 latency (ps>.07). Similarly, the analyses of the frontocentral Nc did not show any significant main effect or interaction (all ps>.56) (Fig. 5).

### 3.2 ERPs evoked at 6 months

**Fig. 3:**
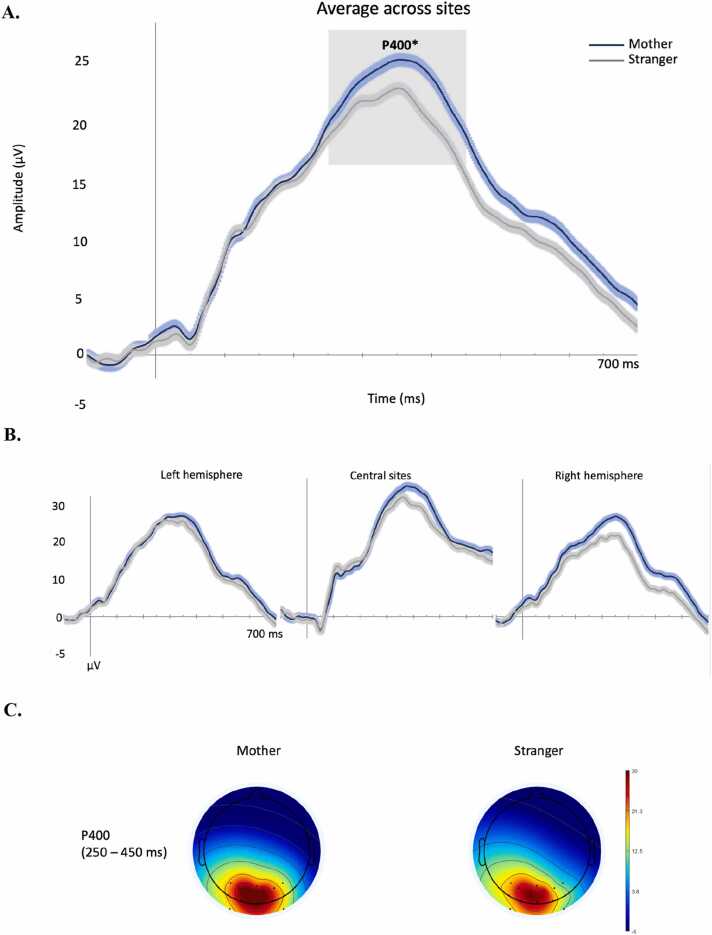
Grand average of the occipitotemporal ERPs evoked by mother and stranger face stimuli in infants at 6 months of age. The time-window selected for the analyses is highlighted in grey. *main effect of face type p < .05. A.: average of ERPs across sites; B.: ERPs over left, right and central sites; C.: topological maps of the P400 time window per face type.

At 6 months, the N290 component was absent, and the face stimuli only elicited a large P400 component (Fig. 3). We analysed its peak amplitude and latency between 250 and 450 ms. The analyses of the peak amplitude revealed an effect of face type, F(1,39)= 4.953, p = .032, η_p_^2^ = .11, showing that the P400 was larger to mother (M = 32.2 μV) than to stranger face stimuli (M = 29.1 μV), and a main effect of location, F(2,78)= 13.479, p < .001, η_p_^2^ = .26, showing larger amplitude over the right sites (M = 26.1 μV) than the left (M= 20.7 μV), t(39) = -3.209, p = .003, and over the central (M= 34.8 μV) than the left, t(39) = 9.338, p < .001, and the right, t(39) = -5.835, p < .001. There was no significant interaction (p = .17). The latency of the P400 was affected by location, F(2,78)= 25.370, p < .001, η_p_^2^ = .39, with an earlier peak over the left (M = 330 ms) and right (M = 337 ms) hemispheres than over the central sites (M = 373 ms), t(39) = -6.211, p < .001, t(39) = -5.646, p < .001, respectively. The effect of face type and the interaction were not significant (ps>.07). The analyses of the frontocentral Nc did not show any significant main effects or interaction of face type and location (ps>.15) (Fig. 5).

### 3.3 ERPs evoked at 9 months

**Fig. 4:**
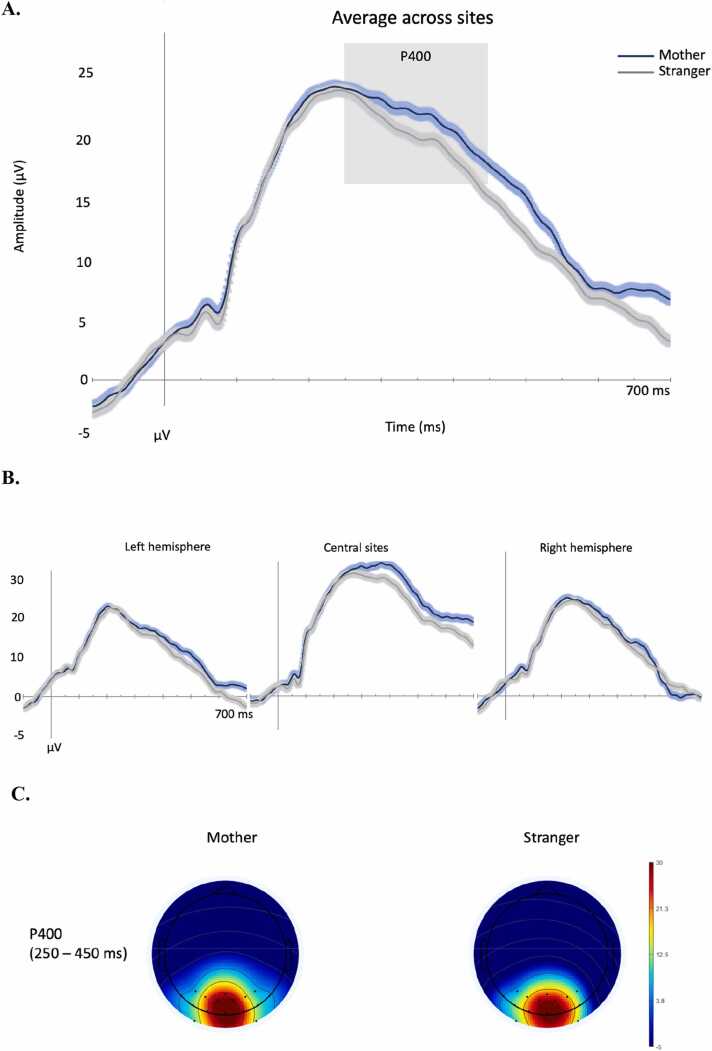
Grand average of the occipitotemporal ERPs evoked by mother and stranger face stimuli in infants at 9 months of age. The time-window selected for the analyses is highlighted in grey. A.: average of ERPs across sites; B.: ERPs over left, right and central sites; C.: topological maps of the P400 time window per face type.

**Fig. 5:**
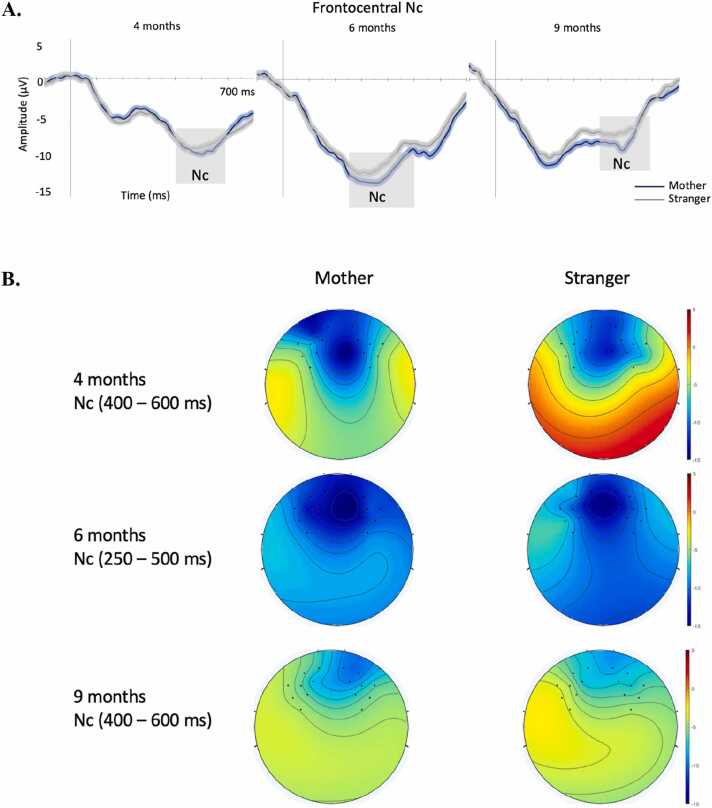
Grand average of the frontocentral Nc evoked by mother and stranger face stimuli in infants at 4, 6, and 9 months of age. The time-windows selected for the analyses are highlighted in grey. A.: average of ERPs across sites; B.: topological maps of the Nc time window at each age per face type.

Similarly to what we observed at 6 months, at 9 months of age the face stimuli only elicited a large P400 component (Fig. 4). The analyses focused on its peak amplitude and latency between 200 and 500 ms. There was an effect of location for the amplitude, F(2,78)= 28.150, p < .001, η_p_^2^ = .42, showing a larger P400 amplitude over the central sites (M = 38.7 μV) than the right (M = 27.8 μV), t(39) = 6.994, p < .001, and the left (M = 28.4 μV), t(39) = 8.240, p < .001. Similarly, there was an effect of location for the P400 latency F(2,78)= 21.175, p < .001, η_p_^2^ = .35, showing a earlier peaks over the right (M = 296 ms) and left (M = 288 ms) sites than centrally (M = 338 ms), t(39) = -5.037, p < .001, t(39) = -5.979, p < .001, respectively. No other significant main effects or interactions were observed (ps>.22). An effect of location was found over the frontocentral Nc, F(2,78)= 4.283, p = .045, η_p_^2^ = .09 (M right = −7.2 μV; M left = −5.6 μV) (Fig. 5). No significant effect of face type or interaction with location were found (all ps>.48).

### 3.4 Developmental trajectory of ERP components

Two Linear Mixed Models (LMM) were run to investigate developmental changes in the amplitude of the P400 and the Nc component, respectively. The LMM for the P400 amplitude revealed a main effect of age, F(2, 211.98)= 26.277, p < .001, η_p_^2^ = .199, driven by a significant amplitude difference between 4 and 6 months, t (216.612) = -8.630, p < .001, Cohen's d = 0.89, and between 4 and 9 months, t (207.432) = -9.942, p < .001, Cohen's d = 0.92. Participants were included as random intercept (variance=.41.65, SD=11.61). The LMM for the Nc amplitude also revealed a main effect of age, F(2, 213.810)= 9.569, p < .001, η_p_^2^ = .08, driven by a significant amplitude difference between 6 and 9 months, t (215.225) = -4.243, p < .001, Cohen's d = 0.66, and a marginal difference between 4 and 6 months, t (218.143) = 2.380, p = .051, Cohen's d = 0.43. Participants were included as random intercept (variance=12.54, SD=4.35). In both LMMs, the main effect of face type and age x face type interaction were non significant (all ps>.14).(Table 1)

**Table 1:** Descriptives of the amplitude (μV) and latency (ms) of the ERP components evoked over left (LH), right (RH) and central sites by the mother and stranger face stimuli at each time point.

			*4 months (N = 38)*		*6 months (N = 40)*		*9 months (N = 40)*	
*ERP component*	*Face type*	*Location*	*Mean (SD)*	Min-Max	*Mean (SD)*	Min -Max	*Mean (SD)*	Min -Max
*N290 amplitude (μV)*	*Mother*	*LH*	2.9 (9.1)	−19.2 – 27.2	-	-	-	-
		*RH*	−1.6 (10.6)	−45.1 – 17.6	-	-	-	-
		*Central*	−2.2 (11.3)	−26.5–22.3	-	-	-	-
	*Stranger*	*LH*	4.7 (9.0)	−16.8 – 24.8	-	-	-	-
		*RH*	1.41 (7.7)	−16.1–20	-	-	-	-
		*Central*	1.21 (10.1)	−21.2 – 29.6	-	-	-	-
*N290 latency (ms)*	*Mother*	*LH*	284 (29.1)	240–333	-	-	-	-
		*RH*	284 (25.4)	241–338	-	-	-	-
		*Central*	261 (25.8)	240–340	-	-	-	-
	*Stranger*	*LH*	285 (25.8)	240–335	-	-	-	-
		*RH*	289 (24.9)	241–335	-	-	-	-
		*Central*	263 (21.4)	240–326	-	-	-	-
*P400 amplitude (μV)*	*Mother*	*LH*	21.2 (9.9)	−3.5 – 44.5	31.7 (13.6)	1.4 – 62.2	28.4 (15.8)	3.1 – 68.6
		*RH*	19.3 (9.6)	−6.1 – 39.7	28.2 (12.2)	10.1 – 64.3	29.2 (13.6)	3.7 – 67.9
		*Central*	24.4 (11.5)	3.9 – 51.2	36.6 (14.8)	12.8 – 79.3	40.4 (15.2)	14.2 – 85.8
	*Stranger*	*LH*	21.9 (11.3)	4.7 – 44.5	30.4 (11.8)	7.5 – 55.3	27.2 (13.4)	3.8 – 59.6
		*RH*	19.4 (7.3)	4.1 – 36.2	23.9 (14.1)	−19.5 – 65.5	27.6 (14.8)	−13.1 – 59.6
		*Central*	24.9 (8.8)	5.5 – 41.2	33 (13.1)	9.6–60.8	37 (14.5)	−.4 – 64.7
*P400 latency (ms)*	*Mother*	*LH*	483 (43.9)	401–600	336 (39.4)	257–411	291 (55.4)	200–399
		*RH*	476 (46.3)	400–597	341 (44.2)	267–433	291 (58.8)	205–418
		*Central*	483 (52.8)	400–600	378 (58.1)	281–592	338 (63.6)	200–467
	*Stranger*	*LH*	481 (47.9)	407–587	323 (43.7)	269–469	286 (58.7)	207–432
		*RH*	475 (49.4)	404–595	334 (43.3)	270–433	300 (54.1)	200–413
		*Central*	494 (51.1)	400–600	368 (44.4)	269–450	337 (74.9)	200–495
*Nc amplitude (μV)*	*Mother*	*LH*	−8.8 (6.2)	−23 – 6.1	−11.6 (6.1)	−22.1 – 5.4	−5.9 (8.3)	−25.2 – 12.2
		*RH*	−7.9 (6.9)	−27.7–11	−12 (6.8)	−24.5 – 6.6	−7.7 (8.4)	−24.4 – 10.8
	*Stranger*	*LH*	−8.7 (5.9)	−21.1 – 8.4	−10.1 (6.6)	−34.6 – 3.1	−5.2 (9.4)	−37.8 – 12.9
		*RH*	−8.3 (4.9)	−16.9 – 7.6	−10.7 (6.6)	−28.9 – 2.5	−6.7 (8.9)	−27.2 – 13.2

### 3.5 Longitudinal associations with falling reactivity

**Fig. 6:**
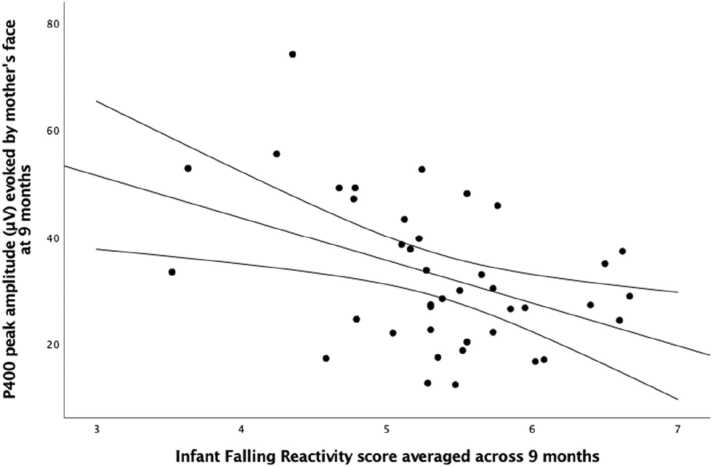
Plot depicting the significant negative correlation between the score of infant falling reactivity assessed with the IBQ-R (Gartstein and Rothbart, 2003) and averaged across 4 time points (2 weeks, 4 months, 6 months and 9 months) and the peak amplitude of the P400 component evoked in response to the mother’s face at 9 months.

Pearson’s correlational analyses were conducted to identify associations between the ERPs evoked by mother and stranger face stimuli reported above and infant falling reactivity as assessed by the infants’ mothers using the IBQ-R (Gartstein and Rothbart, 2003). We first looked at associations between the amplitude of the components elicited by the face stimuli at each age and the average score of infant falling reactivity across the first 9 months of life (Table 2). While the analyses did not reveal any association with the components observed at 4 and 6 months of age, we found a negative association between infant falling reactivity and the P400 amplitude evoked by the mother face at 9 months, r = -.423, p = .007 (Fig. 6). There was also a positive association between infant falling reactivity and Nc amplitude evoked by the mother face at 9 months, r = .353, p = .025, however this did not survive after controlling for multiple comparisons (4 comparisons (2 Face types x 2 ERP components), q= .0125). There were no significant associations between falling reactivity and the ERP components in response to the stranger’s face.

**Table 2:** Correlations between the score of infant falling reactivity assessed with the IBQ-R (Gartstein and Rothbart, 2003) and averaged across four time points (2 weeks, 4 months, 6 months and 9 months) and the visual ERP components (N290, P400, Nc) evoked in response to the mother and a stranger face at 4, 6 and 9 months.

		*Falling Reactivity 0–9 months*
*ERPs at 4 months*	*N290 Mother*	−.141
	*N290 Stranger*	−.117
	*P400 Mother*	−.13
	*P400 Stranger*	−.08
	*Nc Mother*	−.009
	*Nc Stranger*	.069
*ERPs at 6 months*	*P400 Mother*	−.09
	*P400 Stranger*	−.157
	*Nc Mother*	−.177
	*Nc Stranger*	.07
*ERPs at 9 months*	*P400 Mother*	−.423**
	*P400 Stranger*	−.234
	*Nc Mother*	.353*
	*Nc Stranger*	.117

*Note*. Sample size: 38 infants at 4 months; 39 infants at 6 months; 40 infants at 9 months.

* p < .05

** p < .01

**Fig. 7:**
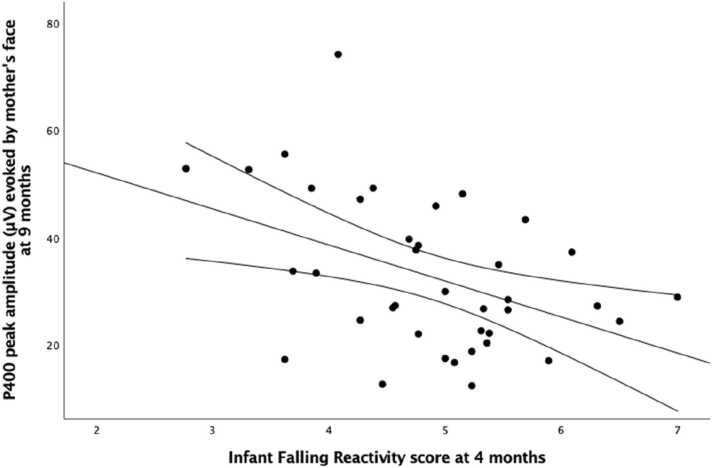
Plot depicting the significant negative correlation between the score of infant falling reactivity measured with the IBQ-R (Gartstein and Rothbart, 2003) at 4 months and the peak amplitude of the P400 component evoked in response to the mother’s face at 9 months.

**Fig. 8:**
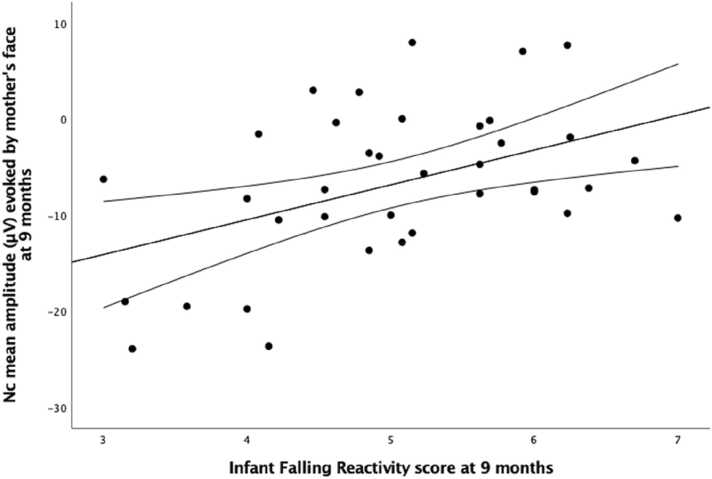
Plot depicting the significant positive correlation between the score of infant falling reactivity at 9 months and the mean amplitude of the Nc component evoked in response to the mother’s face at 9 months.

Given the above associations, exploratory correlational analyses were run to investigate longitudinal associations between falling reactivity at each assessment point (2 weeks, 4 months, 6 months, 9 months) and the amplitude of P400 and Nc elicited by the mother face at 9 months (Table 3). These revealed that only infant falling reactivity reported at 4 months was significantly associated (negative correlation) with the amplitude of P400 elicited by the mother face at 9 months, r = -.435, p = .006 (Fig. 7). (Note that infant falling reactivity at 2 weeks already showed a trend towards a negative correlation with the amplitude of P400 elicited by the mother face at 9 months, r = -.314, p = .055). The amplitude of the Nc elicited by the mother face at 9 months was positively correlated with falling reactivity at 4 and 9 months, r = .377, p = .02 and r = .455, p = .005, respectively. However only the latter association survived after controlling for multiple comparisons (8 comparisons, q=.006) (Fig. 8). Refer to the Supplementary Material for the complete matrix of longitudinal associations between falling reactivity and the amplitude of the ERP components at each age of assessment (Table S2), and for complete matrices of the longitudinal associations between scores on the other temperament scales (distress and negative affect) and the amplitude of the ERP components at each age of assessment (Table S3 and Table S4).

**Table 3:** Correlations between the score of infant falling reactivity assessed with the IBQ-R (Gartstein and Rothbart, 2003) at four time points (2 weeks, 4 months, 6 months and 9 months) and the visual ERP components (P400, Nc) evoked in response to the mother’s face at 9 months.

	*ERPs evoked by the mother face at 9 months*	
	*P400*	*Nc*
*Falling Reactivity at 2 weeks*	−.314	.103
*Falling Reactivity at 4 months*	−.435**	.377*
*Falling Reactivity at 6 months*	−.241	.198
*Falling Reactivity at 9 months*	−.285	.455**

*Note*. Sample size: 38 infants at 2 weeks and at 4 months; 37 infants at 6 months and at 9 months.

* p < .05

** p < .01

## 4 Discussion

With this research, we aimed to investigate i) the longitudinal trajectory of the infant ERP components evoked by the processing of mother and stranger face stimuli during the first year of life, ii) the time points where such components showed significant differences between the face stimuli and iii) how these neural responses related to infant falling reactivity.

### 4.1 Development of face-sensitive ERP components

First, we found that not all of the expected face-sensitive ERP components were elicited by the stimuli after 4 months of age. In fact, while we observed both the occipitotemporal N290 and the P400 components at 4 months, we only observed a P400 component at 6 and 9 months. While this differs from seminal infant ERP work on face processing (e.g., de Haan et al., 2002; Halit et al., 2003), our ERP waveforms closely resemble those reported in Balas et al. (2010) with 6-month-old infants where a N290 was indeed absent. We believe that it is unlikely that the absence of this component is due to stimuli and testing repetition in our longitudinal design since Balas et al. (2010) employed a cross-sectional design instead. However future research should aim to investigate further this developmental change and understand the factors that influence the presence of the face-sensitive components.

For those components that were observed at each time points, the P400 and the Nc, we found an effect of age, indicating that the amplitude of these components change with time. Specifically, we found that the P400 amplitude significantly increased from 4 months, reaching stability at 6 months of age. In contrast, the Nc showed a larger amplitude at 6 months compared to both 4 and 9 months, indicating a U-shaped trajectory. In line with Webb et al. (2005), who also found a non-uniform pattern of Nc response across ages, we concur that these changes may reflect developmental changes in cognitive abilities, or even visual preferences. Indeed, in our behavioural study with the same cohort (Rigato et al., 2023), we report that infants looked significantly longer at their mother’s face (vs a stranger) only at 6 months, but not at 4 or 9 months. In addition, when investigating associations between our behavioural and ERP data, we found a significant correlation between the time infants spend looking at a stranger face at 6 months and the amplitude of the Nc evoked by the stranger face at 9 months (see Table S5 in Supplementary Material for full correlations between the behavioural data collected in (Rigato et al., 2023) and the ERP data of the current manuscript).

Second, our analyses revealed that the face-sensitive ERP components were larger to the mother face than the stranger face at 4 months (N290) and at 6 months (P400), however they no longer showed a significant difference between the two faces at 9 months. Interestingly, and in contrast to what we were expecting based on de Haan and Nelson (de Haan and Nelson, 1997), we did not find any difference between the two stimuli over the frontocentral Nc. This component is thought to reflect processes related to attention and stimulus recognition for information stored in long-term memory (e.g., Courchesne et al., 1981), therefore we had expected to see an enhancement in response to the mother face. Instead, our data seem to indicate that these processes can also pertain to the occipitotemporal components N290 and P400. As suggested by Balas et al. (2010), it is possible that at 6 months the P400 reflects experience with particular faces. Accordingly, our findings would then suggest that exposure to the mother face could also influence face processing earlier on, i.e., at 4 months of age, and also at earlier temporal/processing stages, i.e., between 260 and 290 ms (N290 peak latency). As a consequence, we might think that these face-sensitive components reflect familiarity processes and identity recognition ability. Indeed, the face of the mother is exceptionally familiar to the infant who is in fact able to discriminate her face from that of a stranger from very early on and throughout the first months of life (e.g., Field et al., 1984; Bushnell et al., 1989; Pascalis et al., 1995; Rigato et al., 2023). It would be interesting to test a broader range of familiarity effects in future studies, using stimuli depicting faces of various degrees of familiarity (similarly to Rigato et al., 2024), also in light of the evidence that the adult face-sensitive component N170 is generally not affected by familiarity (Bentin and Deouell, 2000, Eimer, 2000).

Alternatively, or in addition to this, these components might also reflect the emotional value associated with the face. In fact, the mother is also, in most cases, the primary caregiver and the first attachment figure for the infant. It is therefore possible that in the first 6 months of life, specific developmental tasks, such as that of forming an attachment relationship with a primary caregiver, influence the neural processes underlying the visual face system. In line with this hypothesis, Scherf and Scott (2012) argued that developmental changes in face-processing abilities are fundamentally influenced by transitions in age-appropriate developmental tasks or goals. In their work, Scherf and Scott (2012) advanced the idea that the need to form an attachment relationship with a caregiver drives the perceptually difficult computational goal of discriminating between faces. The developmental trajectory of the formation of attachment relationships would therefore be associated with the strength and magnitude of face recognition biases in infancy. Consistent with this expectation, in our work investigating the longitudinal trajectories of visual face preference (Rigato et al., 2023) and face processing at the neural level (the current study) in the first year of life, we report significant differences in both behavioural and neural responses between the mother’s face and a stranger’s face up to 6 months of age. In both cases, we observe that by 9 months of age, infants tend to no longer show a visual preference or an enhanced brain response to their mother’s face vs a stranger’s face. We could speculate that the absence of such specific responses to the mother’s face by this age, which also often coincides with an expansion of the infant’s social environment, is an adaptive response. According to Scherf and Scott (2012) proposal, such reduction of face-processing biases may be the reflection of infants’ developing additional attachment relationships with other individuals (e.g., day care providers, other family members), and of a re-organisation of the face system which will increasingly reflect the perceptual characteristics of these other individuals.

### 4.2 Neural correlates and temperament associations

A final aim of our study was to examine the relationship between infant ERP responses to the mother and stranger face stimuli and falling reactivity. We found that the P400 amplitude evoked by the mother face at 9 months (but not at any of the other ages tested) was negatively associated with infant falling reactivity measured throughout the first year of life (average of scores collected at 2 weeks, 4 months, 6 months and 9 months). As such, infants who were perceived by their mothers as having more difficulty regulating their affect had a larger (i.e., more positive) P400 amplitude in response to the mother’s face at 9 months than those who easily settled down after peak affect. Further analyses revealed that this effect was particularly driven by the score of infant falling reactivity reported by the mother when the infant was 4 months old, as such, infants who displayed lower falling reactivity in early infancy (4 months) later had a larger P400 component in response to their mother’s face (9 months). Furthermore, we found a significant concurrent positive association between the Nc amplitude elicited by the mother’s face and the level of infant falling reactivity reported by the mothers at 9 months. This showed that infants with larger (i.e., more negative) Nc amplitude were scored by their mothers as having more difficulty regulating their affect. Altogether, these results indicate that infants who at 9 months display a smaller P400 and a smaller Nc amplitude in response to the mother’s face, have higher level of falling reactivity.

These findings are important because they indicate that the neural processes associated with the processing of faces, and specifically the face of the mother, are related to infant individual characteristics. In addition, they could also help delineate a typical developmental trajectory where strong responses to the mother’s face present in the first half of the first year of life wane by 9 months of age indicating neural maturity and adaptive emotion regulation skills. In turn, we may speculate that infants who still show a strong neural response (e.g., a large P400) to the mother’s face by the end of the first year are those infants who struggle to regulate their own arousal (i.e., have lower falling reactivity), and perhaps still rely more on the mother for self-regulation. Swingler et al., 2007, Swingler et al., 2010 previously observed that the P400 and the Nc are related to the infant’s social reaction to the mother, and in particular to infant distress during her absence and proximity-seeking behaviours during interactions. While a direct comparison of the effects observed in those studies and in the current report is difficult given the differences in the measures employed, it is important to note that these effects encompassed the same ERP components. With the present study, we extend previous cross-sectional findings relating specifically to behaviours observed during mother-infant interactions (Swingler et al., 2007, Swingler et al., 2010) by showing that the occipitotemporal P400 and the frontocentral Nc components are associated with infant self-regulation across the first 9 months of life. Critically, our longitudinal design allowed to reveal how these associations unfold with time and show an early effect of infant falling reactivity (at 4 months) on later ERP response to the mother’s face (P400 at 9 months) and a concurrent late effect of infant falling reactivity (at 9 months) on the Nc in response to the mother’s face.

### 4.3 Limitations and future research

Our study has a number of limitations. While our findings are novel in terms of showing significant associations between infant brain responses and temperament, we need to acknowledge that the measure of falling reactivity was collected through maternal report. Parent-reported measures are a valuable resource as they allow the assessment of infant self-regulation in daily routine situations across a variety of contexts where parents have more opportunities to observe the infant (Pinto and Figueiredo, 2023), however most of these situations are social by necessity, adding a social component to self-regulation. Therefore, we need to acknowledge that our measure of falling reactivity might also reflect elements of social regulation in addition to self-regulation. It will be crucial to replicate these findings through direct observation of infant behaviour, or to corroborate the maternal reports with another observer (e.g., father, nursery worker, health visitor). Another limitation of the current study is the use of the same stranger face across assessment points. A possible reason for not finding significant differences between the face stimuli over the Nc component might be that the infant became familiar with that stimulus. Future longitudinal research could test whether pairing the mother’s face with a new unfamiliar face at each session would evoke significant Nc differences. However, it is also important to consider that introducing stimulus changes within a longitudinal study design may confound developmental effects. Alternatively, provided that enough trials can be collected, assessing repetition suppression effects would be a good method to address the effect of using the same stranger face across time points. More generally, further work is needed to shed light on what types of self-regulation link to brain responses. It would be interesting to investigate whether the current findings transfer to contexts where self-regulation is needed but does not involve the mother (e.g., in a nursery environment). It would also be important to employ a broader range of self-regulation measures.

Despite these limitations, our present work reveals novel findings regarding the infant face-sensitive ERP components and contributes to the literature on the development of early face processing. Our results suggest that the way the infant’s brain processes the mother’s face is related to the developing self-regulation abilities.

## CRediT authorship contribution statement

**Karla Holmboe:** Writing – review & editing, Supervision, Investigation, Funding acquisition, Conceptualization. **Henrik Dvergsdal:** Writing – review & editing, Software, Methodology. **Manuela Stets:** Writing – review & editing, Project administration, Formal analysis, Data curation. **Silvia Rigato:** Writing – review & editing, Writing – original draft, Supervision, Investigation, Funding acquisition, Formal analysis, Data curation, Conceptualization.

## Declaration of Competing Interest

The authors declare that they have no known competing financial interests or personal relationships that could have appeared to influence the work reported in this paper.

## Data Availability

Data will be made available on request.
