# Supplementary material for: Infant neural processing of mother’s face is associated with falling reactivity in the first year of life
Source: Dev Cogn Neurosci. 2024 Dec 30;71:101502. doi: 10.1016/j.dcn.2024.101502 (PMC11780142; doi:10.1016/j.dcn.2024.101502)
Supplement: Supplementary file 1 — Supplementary material [file mmc1.docx]

**SUPPLEMENTARY MATERIAL**

**Infant neural processing of mother’s face is associated with falling reactivity in the first year of life**

**SUPPLEMEMENTARY METHODS**

*Questionnaire Measures*

Infant temperament was assessed with the IBQ-R VSF (Putnam et al., 2014) and the IBQ-R (Gartstein & Rothbart, 2003). The IBQ-R VSF consists of 37 items in total, and can be separated into three broad scales: Surgency (13 items; e.g., “When tossed around playfully, how often did the baby laugh?”), Negative Affect (12 items; e.g., “At the end of an exciting day, how often did your baby become tearful?”), and Orienting/Regulatory Capacity (12 items; e.g., “How often during the last week did the baby enjoy being read to?”) (Putnam et al., 2014). In addition to these scales, we also included the scales for Distress (12 items; e.g., “After sleeping, how often did the baby fuss or cry immediately?”) and Falling Reactivity (13 items; e.g., “When put down for a nap, how often did your baby settle down quickly?”) from the IBQ-R (Gartstein & Rothbart, 2003). For each question, mothers were asked to rate how often their infant had shown a specific behavior in a given situation during the past week by selecting a score between 1 (“Never”) and 7 (“Always”). Additionally, an item could be scored as “NA – Does not apply” should the situation described in the question not have occurred over the previous seven days. Average scores were calculated for each scale. Items rated as “NA – Does not apply” were excluded from analysis.

Putnam et al. (2014) reported an average Cronbach’s alpha of .75 or above for the three broad IBQ-R VSF scales, and an average Cronbach’s alpha of .78 specifically for the Negative Affect scale. In the current study, Cronbach’s alpha for the Negative Affect scale ranged from .79 to .88 across ages, with a mean alpha of .82. Montirosso et al. (2011) reported average Cronbach’s alphas of .83 and .84 across two samples for the variables of Distress and Falling Reactivity, respectively. An overview of a number of studies using different versions of the IBQ-R revealed that Cronbach’s alpha for Distress and Falling Reactivity ranged between .74 and .79 (*M* = .76) and between .76 and .84 (*M* = .80), respectively, when using the short version of the IBQ-R (Putnam et al., 2014). In the current study, Cronbach’s alpha for the Distress scale ranged from .59 to .71 across ages, with a mean alpha of .65, and Falling Reactivity ranged from .65 to .85 across ages, with a mean alpha of .78.

**MISSING DATA IN THE LINEAR MIXED MODELS**

A total of 59 participants were included in the linear mixed models (LMM). ERP data for each assessment time point (4, 6 and 9 months) were included for 18 participants, while the other 41 participants had some missing points (from 1 or 2 timepoints, Table S1). These were accounted for in the analyses (using maximum likelihood estimation). Information about missing data for each assessment point are also provided in Table S1.

**Table S1.** Participant data included in the LMM per assessment point and missing data

|  | | *4 months* | *6 months* | *9 months* |
| --- | --- | --- | --- | --- |
| *One assessment point* | | 5 | 8 | 5 |
| *Two assessment points* | *4 and 6 months* | 6 | 6 | - |
|  | *4 and 9 months* | 9 | - | 9 |
|  | *6 and 9 months* | - | 8 | 8 |
| *Three assessment points* | | 18 | 18 | 18 |
| *Completed data* | | 38 | 40 | 40 |
| *Missing data* | | 21 (36%) | 19 (32%) | 19 (32%) |

An independent t test was run to compare the family income of those participants who contributed to the data at each point of assessment (N=18, M=£62K, SD=£37K) and those for which we had missing points (N=39, M=£50K, SD=£21K). Two participants did not disclose this information. The t test revealed that the participants did not differ in terms of family income, t(55) = 1.562, p=.124. The same test was run at each assessment point and confirmed that participants did not differ in terms of family income either at 4 months, t(55) = 1.120, p=.268, 6 months, t(55) = 1.009, p=.317, nor 9 months, t(55) = .397, p=.693.

**SUPPLEMENTARY ANALYSES AND RESULTS**

*Associations between ERPs and temperament*

Additional correlational analyses were conducted between the ERP components evoked by the mother’s face at 4, 6 and 9 months and the score of infant falling reactivity and distress measured with the IBQ-R (Gartstein & Rothbart, 2003) and negative affect measured with the IBQ-R VSF (Putnam et al., 2014) as reported by the mother when the infant was 2 weeks, 4 months, 6 months, and 9 months of age.

The analyses showed that falling reactivity at 4 month was associated with the P400 amplitude and the Nc amplitude at 9 months, r=-.435, p=.006 and r=.377, p=.02, respectively. In addition, falling reactivity at 9 months was associated with the Nc amplitude at 9 months, r=.455, p=.005. No other significant correlations were found (Table S2).

While no significant association was found with negative affect (Table S3), the analyses revealed that distress at 6 months positively correlated with the Nc amplitude at 6 months, r=.333, p=.044, and distress at 9 months positively correlated with the P400 amplitude at 4 months, r=.361, p=.033 (Table S5).

**Table S2.** Correlations between falling reactivity as measured with the IBQ-R (Gartstein & Rothbart, 2003) and ERP components evoked by the mother face at each age of assessment.

| *ERPs evoked by mother face* | *4 months* | | | *6 months* | | *9 months* | |
| --- | --- | --- | --- | --- | --- | --- | --- |
|  | *N290* | *P400* | *Nc* | *P400* | *Nc* | *P400* | *Nc* |
| *Falling Reactivity at 2 weeks* | -.077  (35) | -.046  (35) | -.032  (35) | .116  (36) | -.286  (36) | -.314  (38) | .103  (38) |
| *Falling Reactivity at 4 months* | -.164  (37) | -.123  (37) | .015  (37) | .052  (36) | -.07  (36) | **-.435****  (38) | **.377***  (38) |
| *Falling Reactivity at 6 months* | -.218  (37) | -.171  (37) | .085  (37) | .107  (37) | -.132  (37) | -.241  (37) | .198  (37) |
| *Falling Reactivity at 9 months* | -.034  (36) | -.118  (36) | -.042  (36) | .001  (37) | -.003  (37) | -.285  (37) | **.455****  (37) |

*Note.* Sample size is specified in brackets for each association. *p<.05, **p<.01.

**Table S3.** Correlations between negative affect as measured with the Infant Behavior Questionnaire–Revised Very Short Form (IBQ-R VSF; Putnam et al., 2014) and ERP components evoked by the mother face at each age of assessment.

| *ERPs evoked by mother face* | *4 months* | | | *6 months* | | *9 months* | |
| --- | --- | --- | --- | --- | --- | --- | --- |
|  | *N290* | *P400* | *Nc* | *P400* | *Nc* | *P400* | *Nc* |
| *Negative Affect at 2 weeks* | -.012  (36) | -.047  (36) | .014  (36) | -.142  (37) | .021  (37) | .283  (39) | -.172  (39) |
| *Negative Affect at 4 months* | .029  (36) | .266  (36) | .007  (36) | .138  (36) | -.145  (36) | -.145  (38) | .022  (38) |
| *Negative Affect at 6 months* | -.043  (36) | .138  (36) | -.258  (36) | -.127  (37) | .125  (37) | -.197  (37) | .143  (37) |
| *Negative Affect at 9 months* | -.111  (35) | .08  (35) | .178  (35) | -.074  (37) | -.093  (37) | -.213  (37) | -.039  (37) |

*Note.* Sample size is specified in brackets for each association.

**Table S4.** Correlations between distress as measured with the IBQ-R (Gartstein & Rothbart, 2003) and ERP components evoked by the mother face at each age of assessment.

| *ERPs evoked by mother face* | *4 months* | | | *6 months* | | *9 months* | |
| --- | --- | --- | --- | --- | --- | --- | --- |
|  | *N290* | *P400* | *Nc* | *P400* | *Nc* | *P400* | *Nc* |
| *Distress at 2 weeks* | .009  (36) | -.083  (36) | .21  (36) | -.034  (37) | .196  (37) | .278  (39) | -.124  (39) |
| *Distress at 4 months* | -.077  (36) | -.016  (36) | .177  (36) | .299  (36) | .019  (36) | .132  (38) | -.045  (38) |
| *Distress at 6 months* | .115  (36) | .21  (36) | .047  (36) | .038  (37) | **.333***  (37) | -.137  (37) | .154  (37) |
| *Distress at 9 months* | .287  (35) | **.361***  (35) | .038  (35) | .308  (37) | -.148  (37) | .085  (37) | -.18  (37) |

*Note.* Sample size is specified in brackets for each association. *p<.05.

*Associations between ERPs and looking time*

Additional correlational analyses were conducted between the ERP components (amplitude and latency) evoked by the mother’s and stranger’s face at 4, 6 and 9 months and the looking time to the same face stimuli recorded in a separate task during the same testing session (Rigato et al., 2023).

We found positive correlations between looking time to stranger at 4 months and N290 latency at 4 months, r=.348, p=.037, and P400 amplitude to mother face, r=.36, p=.031; looking time to mother at 4 months and P400 latency to stranger were negatively associated, r=-.452, p=.006 (none surviving multiple comparison correction (6x4=24 comparisons, q=.002).

Negative correlations were also found between looking time to mother at 4 months and Nc amplitude at 6 months to the mother face, r=-.437, p=.042, as well as to the stranger face, r=-.458, p=.032 (none surviving multiple comparison correction (6x4=12 comparisons), q=.004).

Finally, a negative correlation was found between looking time to stranger at 6 months and the amplitude of the Nc to stranger at 9 months, r=.605, p=.002.

**Table S5.** Correlations between ERP components and visual looking time to the mother and strager face at each age of assessment.

|  | | | **Looking time**  **at 4 months** | | **Looking time**  **at 6 months** | | **Looking time**  **at 9 months** | |
| --- | --- | --- | --- | --- | --- | --- | --- | --- |
| **ERP componets** | | | *Mother* | *Stranger* | *Mother* | *Stranger* | *Mother* | *Stranger* |
| **4 months** | *N290 amplitude* | *Mother* | -.145 (36) | -.07 (36) | .379 (22) | -.061 (22) | -.028 (21) | -.166 (21) |
|  |  | *Stranger* | -.028 (36) | .035 (36) | .336 (22) | -.099 (22) | .09 (21) | -.064 (21) |
|  | *N290 latency* | *Mother* | -.086 (36) | .348*  (34) | .073 (22) | -.132 (22) | .139 (21) | .015 (21) |
|  |  | *Stranger* | -.09 (36) | .266 (36) | .412 (22) | .036  (22) | .257 (21) | .166 (21) |
|  | *P400 amplitude* | *Mother* | .224 (36) | -.36* (36) | .301 (22) | .326  (22) | -.275 (21) | -.347 (21) |
|  |  | *Stranger* | -.053 (36) | -.261 (36) | .294 (22) | .062 (22) | -.309 (21) | -.419 (21) |
|  | *P400 latency* | *Mother* | -.198 (36) | .026 (36) | -.05 (22) | .096 (22) | -.081 (21) | .074 (21) |
|  |  | *Stranger* | -.452**  (36) | -.309 (36) | -.112 (22) | .088 (22) | -.362 (21) | -.15 (21) |
|  | *Nc amplitude* | *Mother* | .183 (36) | .312 (36) | -.377 (22) | -.192 (22) | .102 (21) | .314 (21) |
|  |  | *Stranger* | .187 (36) | .143 (36) | -.282 (22) | -.19  (22) | .254 (21) | .135 (21) |
| **6 months** | *P400 amplitude* | *Mother* | .169 (22) | -.121 (22) | .08 (35) | .037  (35) | -.104 (19) | -.024 (19) |
|  |  | *Stranger* | .229 (22) | .151 (22) | .083 (35) | .094  (35) | .03  (19) | -.123 (19) |
|  | *P400 latency* | *Mother* | -.245 (22) | -.218 (22) | -.136 (35) | .075  (35) | .032 (19) | .064 (19) |
|  |  | *Stranger* | .052 (22) | .111 (22) | -.05 (35) | .248  (35) | .445 (19) | -.311 (19) |
|  | *Nc amplitude* | *Mother* | -.437* (22) | .113 (22) | .002 (35) | -.106 (35) | -.054 (19) | .204 (19) |
|  |  | *Stranger* | -.458* (22) | .05  (22) | -.035 (35) | -.142 (35) | -.036 (19) | .449 (19) |
| **9 months** | *P400 amplitude* | *Mother* | -.014 (25) | .134 (25) | .071 (23) | .105  (23) | .013 (31) | -.231 (31) |
|  |  | *Stranger* | .094 (25) | .003 (25) | .233 (23) | .364  (23) | .105 (31) | -.287 (31) |
|  | *P400 latency* | *Mother* | -.012 (25) | -.25 (25) | -.082 (23) | -.076 (23) | -.021 (31) | .158 (31) |
|  |  | *Stranger* | .096 (25) | -.315 (25) | -.038 (23) | .241  (23) | -.013 (31) | .269 (31) |
|  | *Nc amplitude* | *Mother* | -.122 (25) | -.058 (25) | .123 (23) | -.311 (23) | -.021 (31) | .261 (31) |
|  |  | *Stranger* | -.334 (25) | .174 (25) | -.111 (23) | **-.605** (23)** | .02  (31) | .147 (31) |

*Note.* Sample size is specified in brackets for each association. *p<.05, **p<.01.

**SUPPLEMENTARY GRAPHS**

We have added below graphs showing the trajectory of the ERP components amplitude per participant evoked by the mother and the stranger face stimuli per region of interest (Figures S1-S6).

**Figure S1.** Trajectory of the N290 amplitude evoked at 4 months of age over the left, central and right occipital sites.
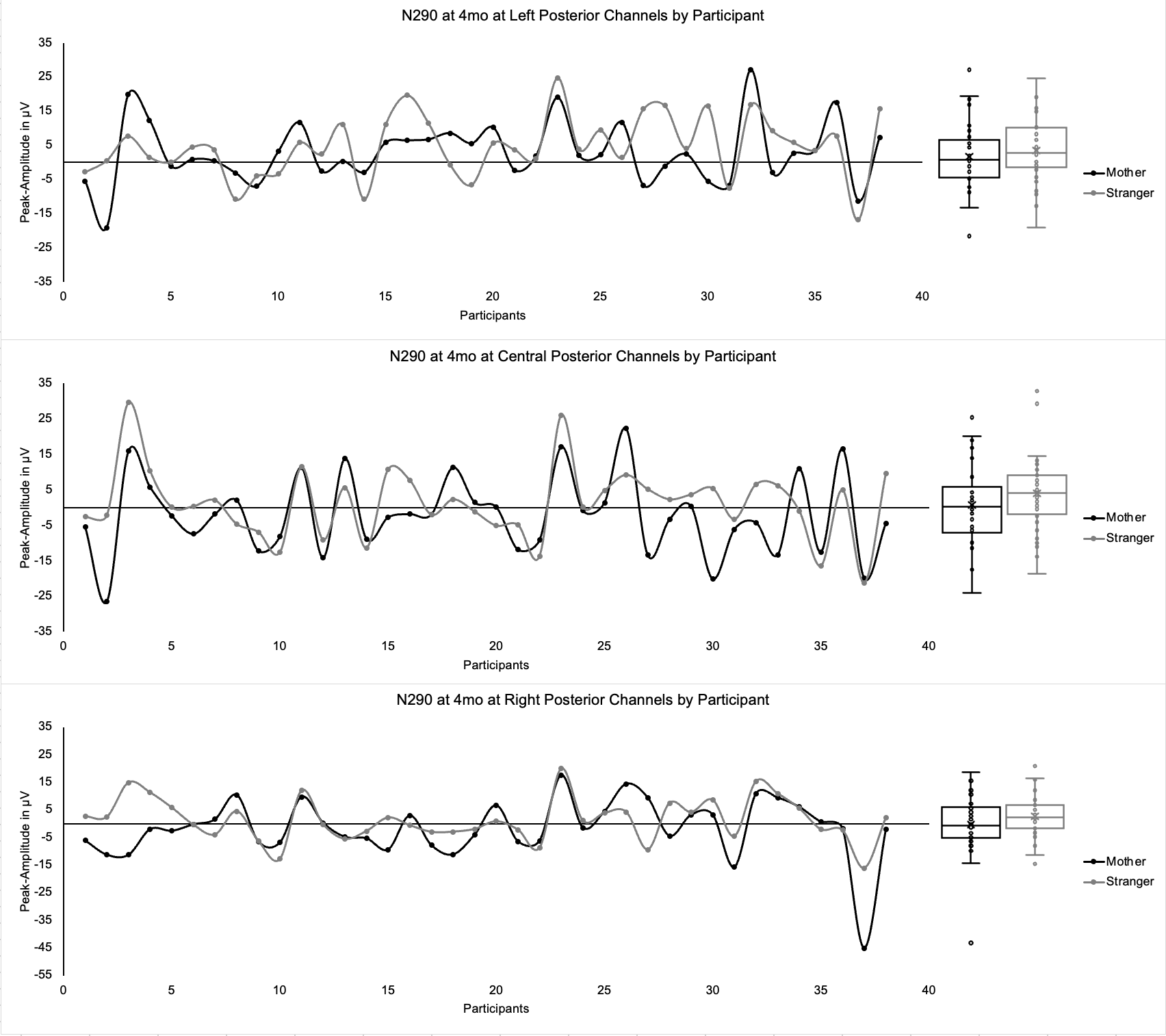


**Figure S2.** Trajectory of the P400 amplitude evoked at 4, 6 and 9 months of age over the left occipital sites.


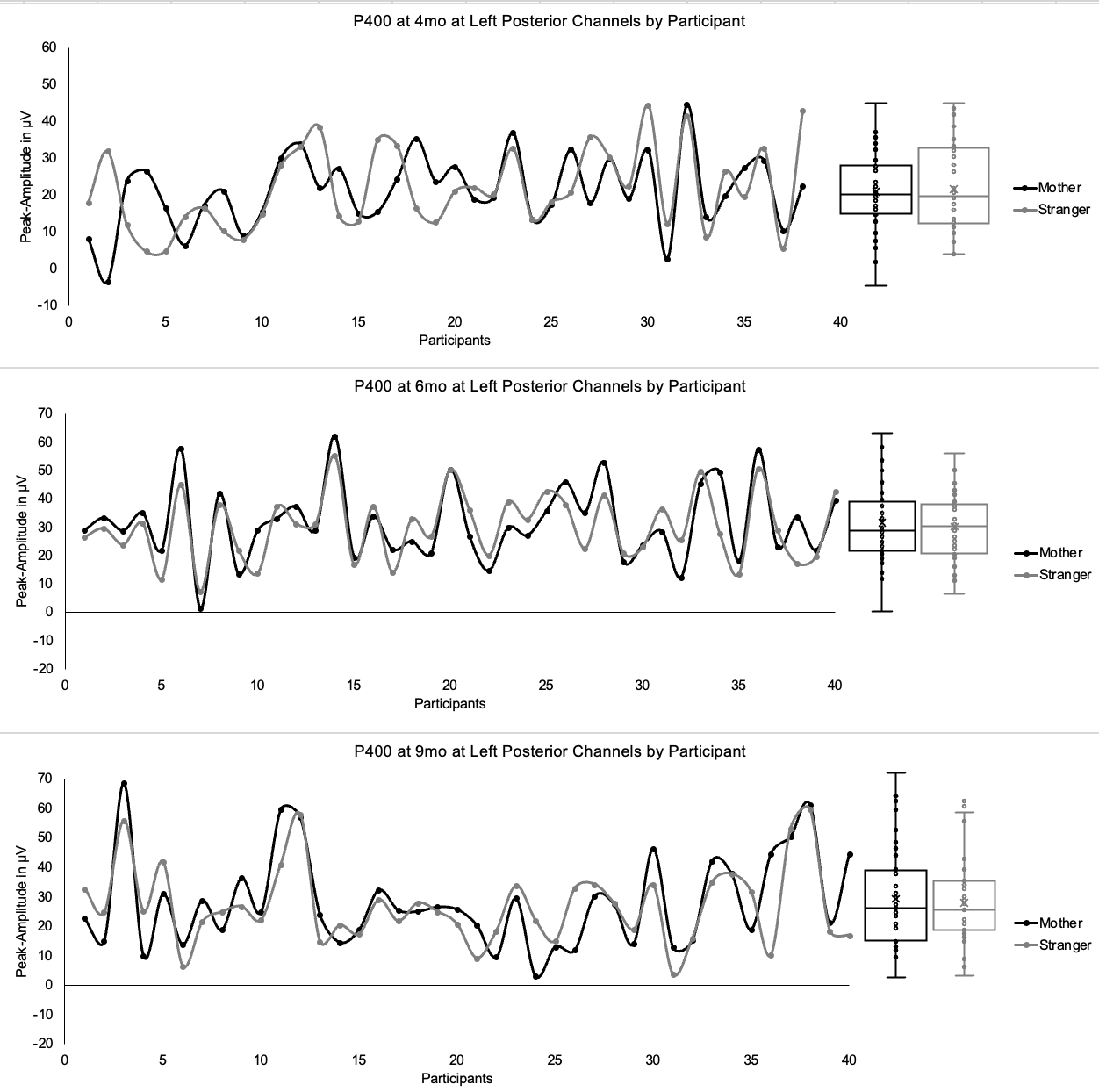


**Figure S3.** Trajectory of the P400 amplitude evoked at 4, 6 and 9 months of age over the central occipital sites.


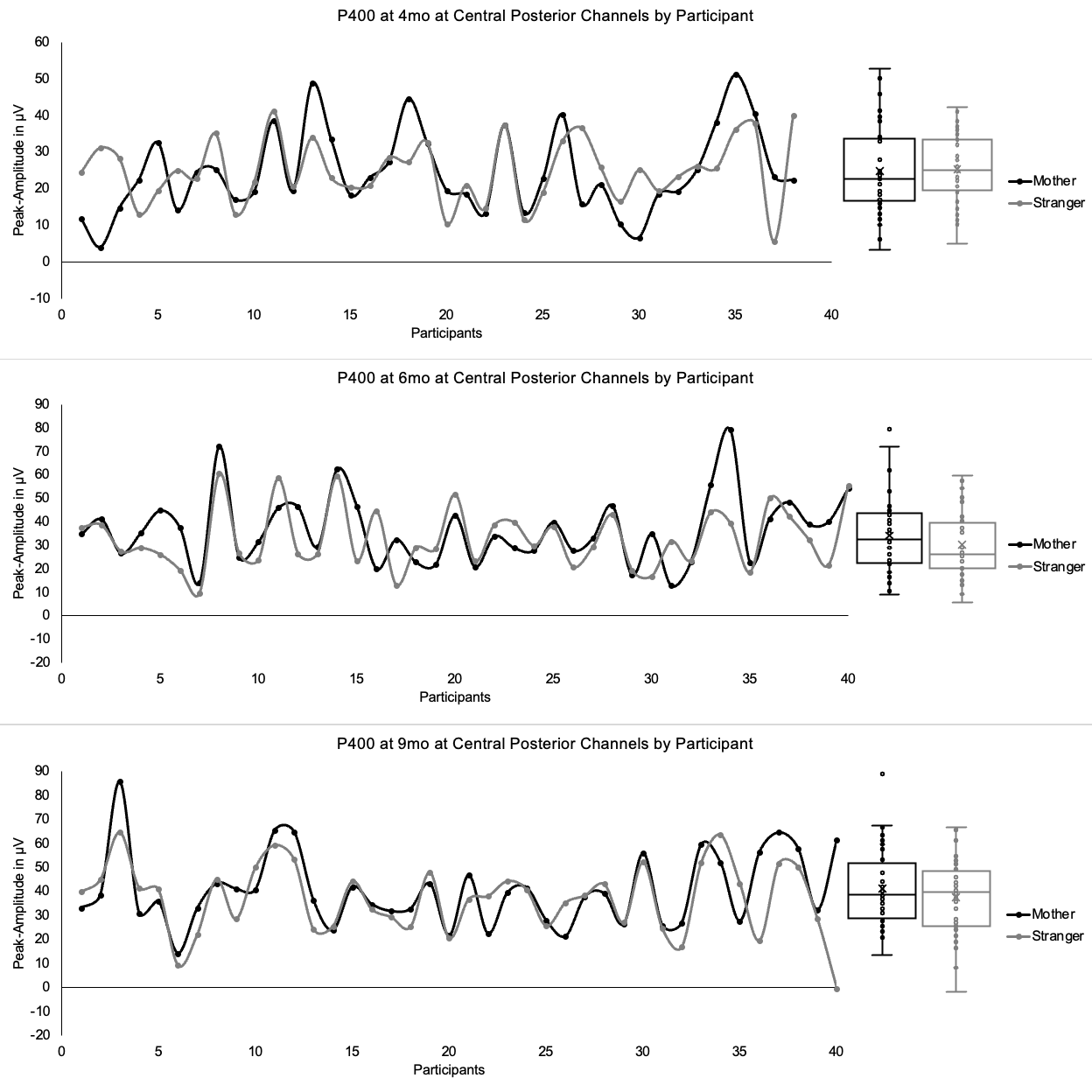


**Figure S4.** Trajectory of the P400 amplitude evoked at 4, 6 and 9 months of age over the right occipital sites.


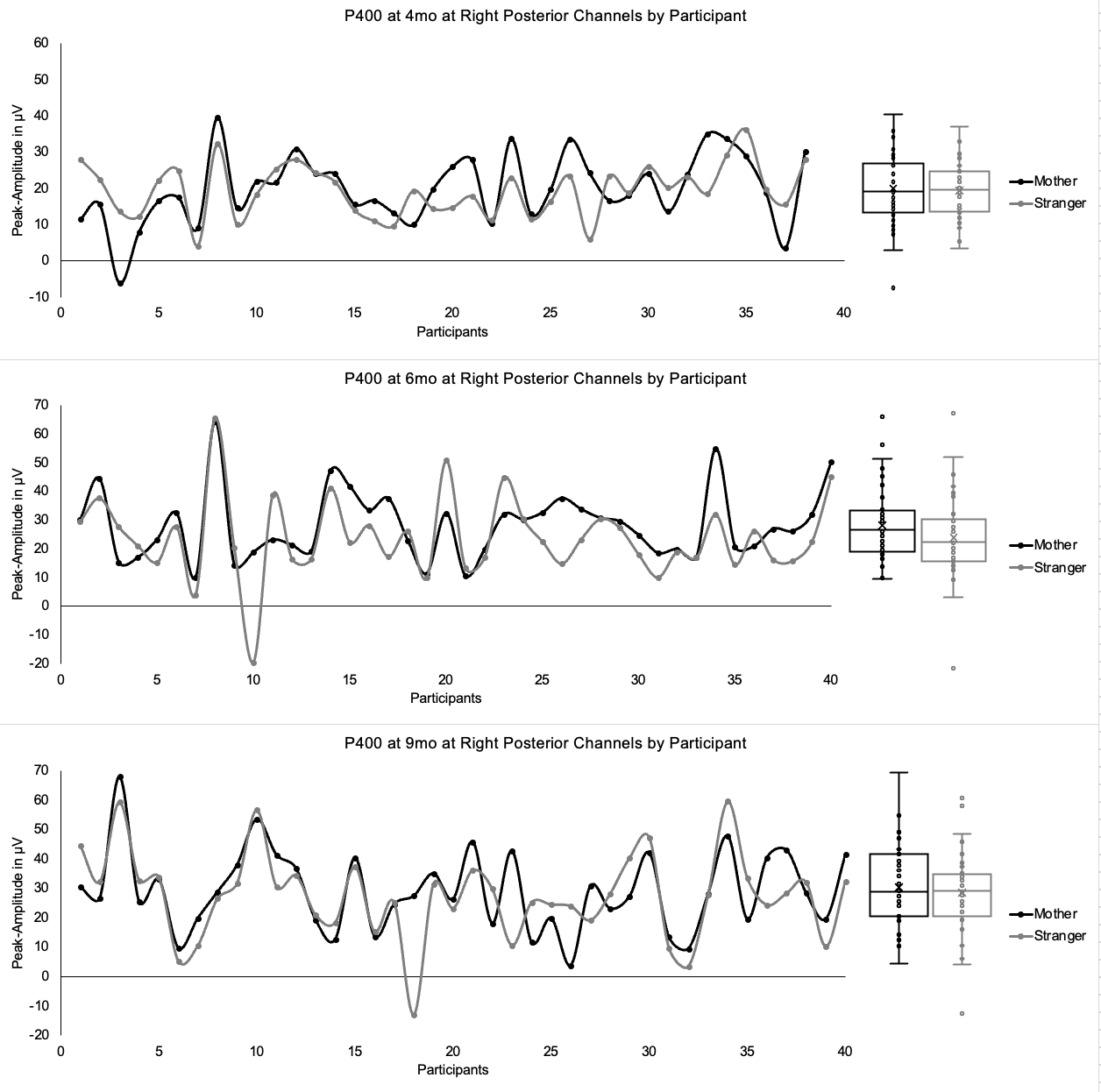


**Figure S5.** Trajectory of the Nc amplitude evoked at 4, 6 and 9 months of age over the left fronto-central sites.


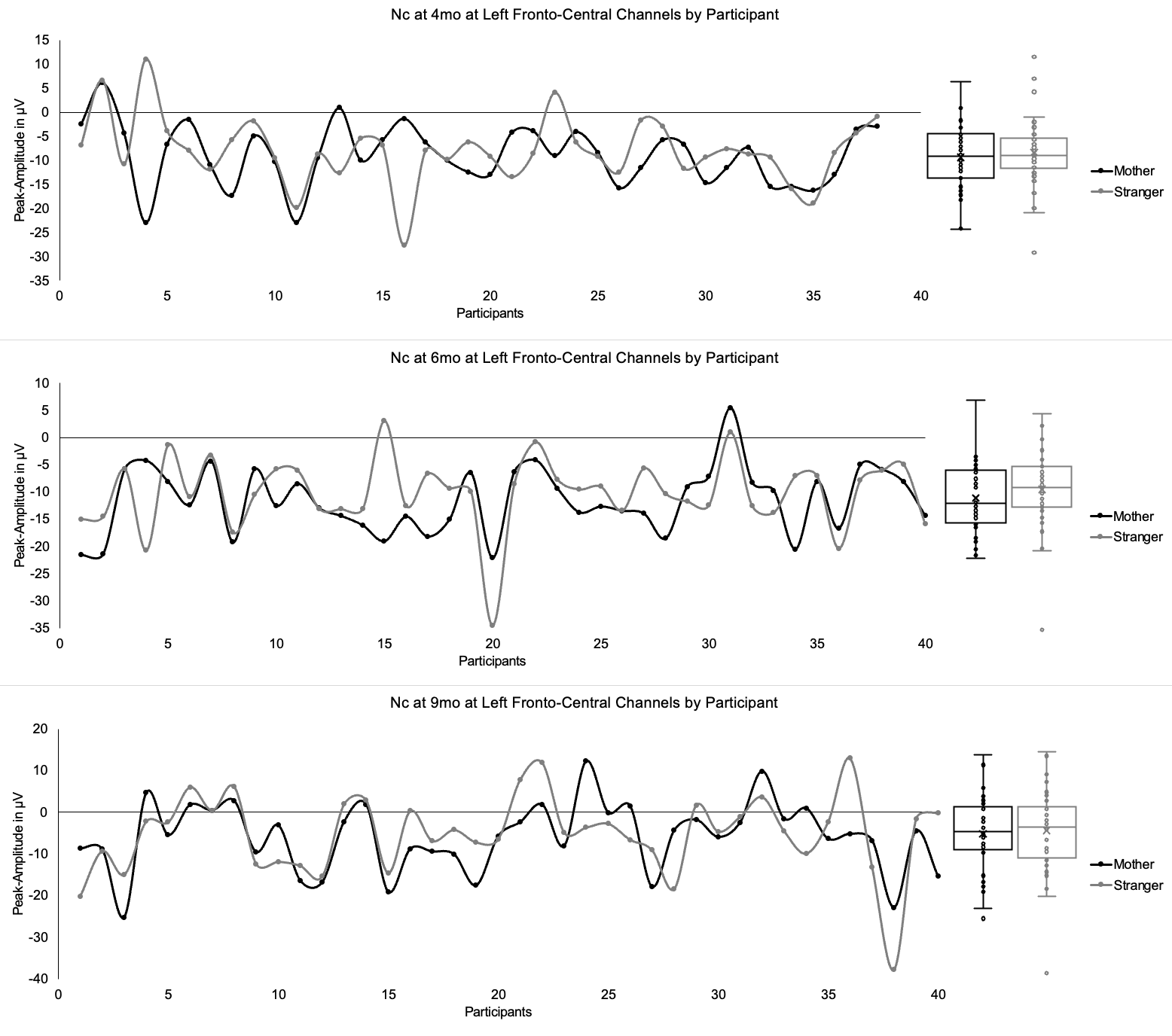


**Figure S6.** Trajectory of the Nc amplitude evoked at 4, 6 and 9 months of age over the right fronto-central sites.

**
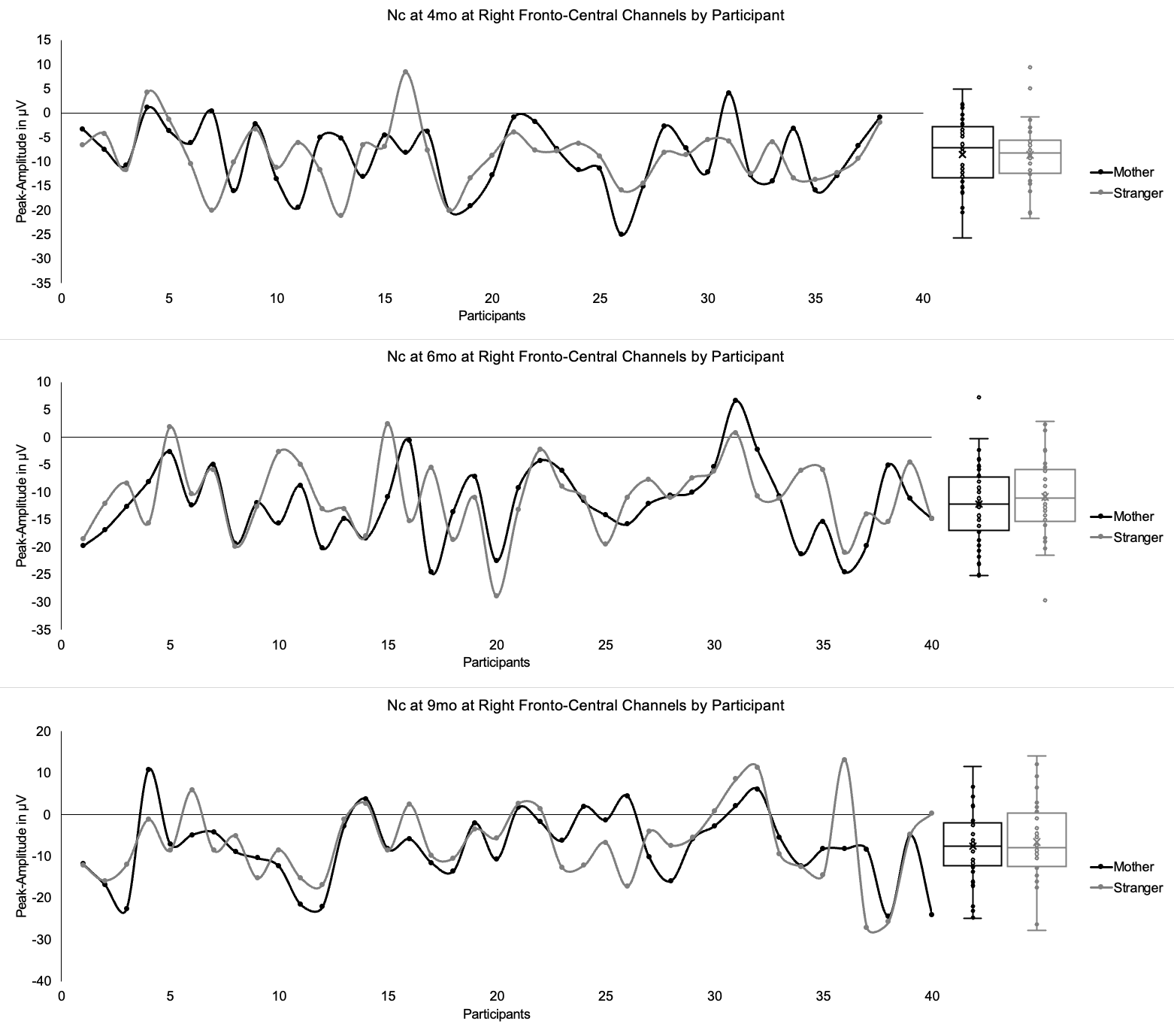
**

**REFERENCES**

Gartstein, M. A. & Rothbart, M. K. (2003). Studying infant temperament via the revised Infant Behavior Questionnaire. Infant Behavior and Development, 26(1), 64–86.

Montirosso, R., Cozzi, P., Putnam, S. P., Gartstein, M. A., & Borgatti, R. (2011). Studying cross-cultural differences in temperament in the first year of life: United States and Italy. International Journal of Behavioral Development, 35(1), 27-37.

Putnam, S. P., Helbig, A. L., Gartstein, M. A., Rothbart, M. K., & Leerkes, E. (2014). Development and Assessment of Short and very Short Forms of the Infant Behavior Questionnaire-Revised. Journal of Personality Assessment, 1-14.

Rigato, S., Stets, M., Charalambous, S., Dvergsdal, H., & Holmboe, K. (2023). Infant visual preference for the mother’s face and longitudinal associations with emotional reactivity in the first year of life. *Scientific Reports, 13*(1), 10263.
